# Dipyridinium tribromidochloridobis(4-chloro­phen­yl)stannate(IV)

**DOI:** 10.1107/S1600536809016687

**Published:** 2009-05-14

**Authors:** Kong Mun Lo, Seik Weng Ng

**Affiliations:** a Department of Chemistry, University of Malaya, 50603 Kuala Lumpur, Malaysia

## Abstract

The tin atom in the  substituted ammonium stannate(IV), (C_5_H_6_N)_2_[SnBr_3_(C_6_H_4_Cl)_2_Cl], lies on a center of symmetry in a distorted octa­hedral coordination geometry. Each independent halogen site is occupied by bromine and chlorine anions in an approximate 3:1 ratio. The pyridinium cation forms a hydrogen bond to only one of the halogen atoms.

## Related literature

For bis­(4-dimethyl­amino­pyridinium) tetra­halido­diorgano­stannates, see: Lo & Ng (2008*a* ▶,*b* ▶); Yap *et al.* (2008 ▶).
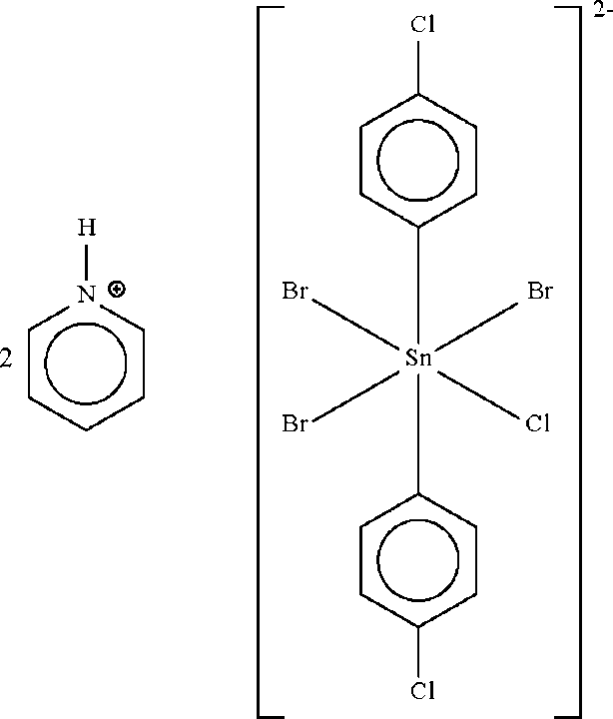


## Experimental

### 

#### Crystal data

(C_5_H_6_N)_2_[SnBr_3_(C_6_H_4_Cl)_2_Cl]
*M* *_r_* = 777.17
Monoclinic, 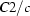

*a* = 11.5130 (2) Å
*b* = 11.7139 (2) Å
*c* = 18.7748 (3) Å
β = 93.230 (1)°
*V* = 2527.99 (7) Å^3^
*Z* = 4
Mo *K*α radiation
μ = 6.08 mm^−1^
*T* = 100 K
0.27 × 0.19 × 0.12 mm

#### Data collection

Bruker SMART APEX diffractometer
Absorption correction: multi-scan (*SADABS*; Sheldrick, 1996 ▶) *T*_min_ = 0.327, *T*_max_ = 0.529 (expected range = 0.298–0.482)
11728 measured reflections
2903 independent reflections
2668 reflections with *I* > 2σ(*I*)
*R*_int_ = 0.022

#### Refinement

*R*[*F* ^2^ > 2σ(*F* ^2^)] = 0.018
*wR*(*F* ^2^) = 0.047
*S* = 1.02
2903 reflections
146 parameters
4 restraints
H-atom parameters constrained
Δρ_max_ = 0.39 e Å^−3^
Δρ_min_ = −0.84 e Å^−3^

### 

Data collection: *APEX2* (Bruker, 2007 ▶); cell refinement: *SAINT* (Bruker, 2007 ▶); data reduction: *SAINT*; program(s) used to solve structure: *SHELXS97* (Sheldrick, 2008 ▶); program(s) used to refine structure: *SHELXL97* (Sheldrick, 2008 ▶); molecular graphics: *X-SEED* (Barbour, 2001 ▶); software used to prepare material for publication: *publCIF* (Westrip, 2009 ▶).

## Supplementary Material

Crystal structure: contains datablocks global, I. DOI: 10.1107/S1600536809016687/bt2945sup1.cif

Structure factors: contains datablocks I. DOI: 10.1107/S1600536809016687/bt2945Isup2.hkl

Additional supplementary materials:  crystallographic information; 3D view; checkCIF report

## Figures and Tables

**Table 1:** Selected bond lengths (Å) (*X* = Br, Cl)

Sn1—C1	2.149 (2)
Sn1—*X*1	2.7166 (2)
Sn1—*X*2	2.7060 (2)

**Fig. 1.**
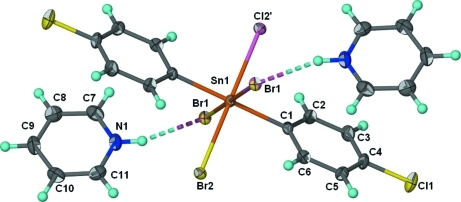
70% Probability anisotropic displacement ellipsoid plot of the ion-pair 2(C5H6 N) [SnBr3Cl(C6H4Cl)2]. Hydrogen atoms are drawn as spheres of arbitrary radius. Dashed lines denote hydrogen bonds. The tin-bound halogen atoms are disordered.

## supplementary crystallographic information

### Experimental

Bis(4-chlorophenyl)tin dichloride (0.40 g, 1 mol) and pyridine hydrobromide perbromide (0.64 g, 2 mmol) were heated in chloroform for 3 h. Crystals separated from the cool solution after a day.

### Refinement

Hydrogen atoms were placed in calculated positions (C—H 0.95, N–H 0.88 Å) and were included in the refinement in the riding model approximation, with *U*(H) set to 1.2*U*(*C*,*N*).

Each of the two independent tin-bound halogen atoms is a mixture of chlorine and bromine; as the total occupancy of chlorine refined to nearly 0.5 and that of bromine to nearly 1.5, these values were fixed as 0.5 and 1.5. Furthermore, the different halogen atoms sharing the same site were constrained to have the same coordinates and the same anisotropic displacement parameters. The final difference Fourier map did not have large peaks/deep holes near the disordered atoms.

### Crystal data

**Table:**

(C_5_H_6_N)_2_[SnBr_3_(C_6_H_4_Cl)_2_Cl]	*F*(000) = 1488
*M**_r_* = 777.17	*D*_x_ = 2.042 Mg m^−^^3^
Monoclinic, *C*2/*c*	Mo *K*α radiation, λ = 0.71073 Å
Hall symbol: -C 2yc	Cell parameters from 6664 reflections
*a* = 11.5130 (2) Å	θ = 2.5–28.3°
*b* = 11.7139 (2) Å	µ = 6.08 mm^−^^1^
*c* = 18.7748 (3) Å	*T* = 100 K
β = 93.230 (1)°	Prism, brown
*V* = 2527.99 (7) Å^3^	0.27 × 0.19 × 0.12 mm
*Z* = 4	

### Data collection

**Table:**

Bruker SMART APEX diffractometer	2903 independent reflections
Radiation source: fine-focus sealed tube	2668 reflections with *I* > 2σ(*I*)
graphite	*R*_int_ = 0.022
ω scans	θ_max_ = 27.5°, θ_min_ = 2.2°
Absorption correction: multi-scan (*SADABS*; Sheldrick, 1996)	*h* = −14→14
*T*_min_ = 0.327, *T*_max_ = 0.529	*k* = −15→15
11728 measured reflections	*l* = −23→24

### Refinement

**Table:**

Refinement on *F*^2^	Primary atom site location: structure-invariant direct methods
Least-squares matrix: full	Secondary atom site location: difference Fourier map
*R*[*F*^2^ > 2σ(*F*^2^)] = 0.018	Hydrogen site location: inferred from neighbouring sites
*wR*(*F*^2^) = 0.047	H-atom parameters constrained
*S* = 1.02	*w* = 1/[σ^2^(*F*_o_^2^) + (0.0246*P*)^2^ + 3.4843*P*] where *P* = (*F*_o_^2^ + 2*F*_c_^2^)/3
2903 reflections	(Δ/σ)_max_ = 0.001
146 parameters	Δρ_max_ = 0.39 e Å^−^^3^
4 restraints	Δρ_min_ = −0.83 e Å^−^^3^

### Fractional atomic coordinates and isotropic or equivalent isotropic displacement parameters (Å^2^)

**Table:**

	*x*	*y*	*z*	*U*_iso_*/*U*_eq_	Occ. (<1)
Sn1	0.5000	0.5000	0.5000	0.01196 (6)	
Br1	0.308690 (19)	0.509165 (19)	0.578153 (12)	0.01383 (8)	0.7365 (11)
Br2	0.551744 (19)	0.288797 (18)	0.550827 (12)	0.01450 (7)	0.7635 (11)
Cl1'	0.308690 (19)	0.509165 (19)	0.578153 (12)	0.01383 (8)	0.2635 (11)
Cl2'	0.551744 (19)	0.288797 (18)	0.550827 (12)	0.01450 (7)	0.2365 (11)
Cl1	0.83429 (5)	0.71755 (5)	0.76748 (3)	0.02688 (12)	
N1	0.15868 (16)	0.59124 (16)	0.43101 (10)	0.0217 (4)	
H1	0.2206	0.5891	0.4607	0.026*	
C1	0.60325 (16)	0.57206 (16)	0.58772 (10)	0.0129 (4)	
C2	0.60187 (18)	0.52532 (17)	0.65598 (11)	0.0165 (4)	
H2	0.5522	0.4626	0.6644	0.020*	
C3	0.67258 (18)	0.56972 (18)	0.71184 (11)	0.0188 (4)	
H3	0.6716	0.5379	0.7584	0.023*	
C4	0.74442 (17)	0.66121 (18)	0.69830 (11)	0.0182 (4)	
C5	0.74654 (17)	0.70998 (17)	0.63157 (11)	0.0171 (4)	
H5	0.7961	0.7729	0.6234	0.021*	
C6	0.67469 (17)	0.66515 (17)	0.57641 (11)	0.0155 (4)	
H6	0.6745	0.6987	0.5303	0.019*	
C7	0.0884 (2)	0.50083 (18)	0.42776 (13)	0.0225 (5)	
H7	0.1048	0.4366	0.4575	0.027*	
C8	−0.0077 (2)	0.50100 (18)	0.38125 (13)	0.0235 (5)	
H8	−0.0579	0.4366	0.3777	0.028*	
C9	−0.03042 (19)	0.5968 (2)	0.33953 (12)	0.0238 (5)	
H9	−0.0971	0.5988	0.3074	0.029*	
C10	0.0440 (2)	0.68937 (19)	0.34465 (12)	0.0231 (5)	
H10	0.0290	0.7552	0.3161	0.028*	
C11	0.13982 (19)	0.68517 (19)	0.39135 (12)	0.0224 (5)	
H11	0.1920	0.7479	0.3955	0.027*	

### Atomic displacement parameters (Å^2^)

**Table:**

	*U*^11^	*U*^22^	*U*^33^	*U*^12^	*U*^13^	*U*^23^
Sn1	0.01296 (10)	0.01329 (10)	0.00948 (10)	−0.00098 (6)	−0.00070 (7)	0.00034 (7)
Br1	0.01297 (12)	0.01610 (12)	0.01249 (13)	−0.00044 (8)	0.00142 (9)	0.00093 (8)
Br2	0.01749 (12)	0.01242 (11)	0.01334 (12)	0.00098 (8)	−0.00136 (8)	0.00188 (8)
Cl1'	0.01297 (12)	0.01610 (12)	0.01249 (13)	−0.00044 (8)	0.00142 (9)	0.00093 (8)
Cl2'	0.01749 (12)	0.01242 (11)	0.01334 (12)	0.00098 (8)	−0.00136 (8)	0.00188 (8)
Cl1	0.0254 (3)	0.0349 (3)	0.0193 (3)	−0.0073 (2)	−0.0073 (2)	−0.0056 (2)
N1	0.0177 (9)	0.0277 (10)	0.0190 (9)	0.0050 (7)	−0.0038 (7)	−0.0047 (8)
C1	0.0126 (9)	0.0155 (9)	0.0103 (9)	0.0020 (7)	−0.0011 (7)	−0.0014 (7)
C2	0.0160 (9)	0.0177 (9)	0.0156 (10)	−0.0017 (7)	0.0004 (8)	−0.0001 (8)
C3	0.0221 (10)	0.0222 (10)	0.0119 (9)	0.0010 (8)	−0.0016 (8)	0.0022 (8)
C4	0.0153 (10)	0.0237 (10)	0.0150 (10)	0.0002 (8)	−0.0041 (8)	−0.0054 (8)
C5	0.0155 (9)	0.0170 (9)	0.0188 (10)	−0.0028 (7)	0.0002 (8)	−0.0013 (8)
C6	0.0154 (9)	0.0170 (9)	0.0142 (10)	0.0012 (7)	0.0018 (7)	0.0010 (8)
C7	0.0251 (12)	0.0223 (11)	0.0204 (12)	0.0064 (8)	0.0055 (9)	0.0023 (9)
C8	0.0194 (11)	0.0239 (11)	0.0276 (13)	−0.0015 (8)	0.0062 (9)	−0.0024 (9)
C9	0.0173 (10)	0.0332 (12)	0.0204 (11)	0.0049 (9)	−0.0028 (8)	−0.0028 (9)
C10	0.0280 (11)	0.0214 (10)	0.0201 (11)	0.0058 (9)	0.0029 (9)	0.0019 (9)
C11	0.0251 (11)	0.0196 (10)	0.0225 (11)	−0.0018 (8)	0.0034 (9)	−0.0048 (9)

### Geometric parameters (Å, °)

**Table:**

Sn1—C1^i^	2.149 (2)	C3—C4	1.386 (3)
Sn1—C1	2.149 (2)	C3—H3	0.9500
Sn1—Br1	2.7166 (2)	C4—C5	1.378 (3)
Sn1—Cl2'^i^	2.7060 (2)	C5—C6	1.391 (3)
Sn1—Br2^i^	2.7060 (2)	C5—H5	0.9500
Sn1—Br2	2.7060 (2)	C6—H6	0.9500
Sn1—Cl1'^i^	2.7166 (2)	C7—C8	1.370 (3)
Sn1—Br1^i^	2.7166 (2)	C7—H7	0.9500
Cl1—C4	1.744 (2)	C8—C9	1.384 (3)
N1—C7	1.332 (3)	C8—H8	0.9500
N1—C11	1.339 (3)	C9—C10	1.383 (3)
N1—H1	0.8800	C9—H9	0.9500
C1—C6	1.389 (3)	C10—C11	1.371 (3)
C1—C2	1.394 (3)	C10—H10	0.9500
C2—C3	1.392 (3)	C11—H11	0.9500
C2—H2	0.9500		
			
C1^i^—Sn1—C1	180.000 (1)	C6—C1—Sn1	119.86 (14)
C1^i^—Sn1—Cl2'^i^	89.29 (5)	C2—C1—Sn1	121.05 (14)
C1—Sn1—Cl2'^i^	90.71 (5)	C3—C2—C1	120.63 (19)
C1^i^—Sn1—Br2^i^	89.29 (5)	C3—C2—H2	119.7
C1—Sn1—Br2^i^	90.71 (5)	C1—C2—H2	119.7
Cl2'^i^—Sn1—Br2^i^	0.000 (13)	C4—C3—C2	118.71 (19)
C1^i^—Sn1—Br2	90.71 (5)	C4—C3—H3	120.6
C1—Sn1—Br2	89.29 (5)	C2—C3—H3	120.6
Cl2'^i^—Sn1—Br2	180.0	C5—C4—C3	121.90 (19)
Br2^i^—Sn1—Br2	180.0	C5—C4—Cl1	118.68 (16)
C1^i^—Sn1—Cl1'^i^	90.05 (5)	C3—C4—Cl1	119.41 (17)
C1—Sn1—Cl1'^i^	89.95 (5)	C4—C5—C6	118.67 (19)
Cl2'^i^—Sn1—Cl1'^i^	90.845 (7)	C4—C5—H5	120.7
Br2^i^—Sn1—Cl1'^i^	90.845 (7)	C6—C5—H5	120.7
Br2—Sn1—Cl1'^i^	89.155 (7)	C5—C6—C1	120.98 (19)
C1^i^—Sn1—Br1^i^	90.05 (5)	C5—C6—H6	119.5
C1—Sn1—Br1^i^	89.95 (5)	C1—C6—H6	119.5
Cl2'^i^—Sn1—Br1^i^	90.845 (7)	N1—C7—C8	119.7 (2)
Br2^i^—Sn1—Br1^i^	90.845 (7)	N1—C7—H7	120.2
Br2—Sn1—Br1^i^	89.155 (7)	C8—C7—H7	120.2
Cl1'^i^—Sn1—Br1^i^	0.000 (8)	C7—C8—C9	118.8 (2)
C1^i^—Sn1—Br1	89.95 (5)	C7—C8—H8	120.6
C1—Sn1—Br1	90.05 (5)	C9—C8—H8	120.6
Cl2'^i^—Sn1—Br1	89.155 (7)	C8—C9—C10	120.0 (2)
Br2^i^—Sn1—Br1	89.155 (7)	C8—C9—H9	120.0
Br2—Sn1—Br1	90.845 (7)	C10—C9—H9	120.0
Cl1'^i^—Sn1—Br1	180.0	C11—C10—C9	119.3 (2)
Br1^i^—Sn1—Br1	180.0	C11—C10—H10	120.3
C7—N1—C11	123.26 (19)	C9—C10—H10	120.3
C7—N1—H1	118.4	N1—C11—C10	119.0 (2)
C11—N1—H1	118.4	N1—C11—H11	120.5
C6—C1—C2	119.08 (18)	C10—C11—H11	120.5
			
Cl2'^i^—Sn1—C1—C6	−40.72 (15)	C1—C2—C3—C4	−0.1 (3)
Br2^i^—Sn1—C1—C6	−40.72 (15)	C2—C3—C4—C5	0.9 (3)
Br2—Sn1—C1—C6	139.28 (15)	C2—C3—C4—Cl1	−179.64 (16)
Cl1'^i^—Sn1—C1—C6	50.12 (15)	C3—C4—C5—C6	−0.4 (3)
Br1^i^—Sn1—C1—C6	50.12 (15)	Cl1—C4—C5—C6	−179.89 (15)
Br1—Sn1—C1—C6	−129.88 (15)	C4—C5—C6—C1	−0.9 (3)
Cl2'^i^—Sn1—C1—C2	140.70 (15)	C2—C1—C6—C5	1.7 (3)
Br2^i^—Sn1—C1—C2	140.70 (15)	Sn1—C1—C6—C5	−176.91 (15)
Br2—Sn1—C1—C2	−39.30 (15)	C11—N1—C7—C8	1.0 (3)
Cl1'^i^—Sn1—C1—C2	−128.46 (15)	N1—C7—C8—C9	−1.2 (3)
Br1^i^—Sn1—C1—C2	−128.46 (15)	C7—C8—C9—C10	0.8 (3)
Br1—Sn1—C1—C2	51.54 (15)	C8—C9—C10—C11	−0.1 (3)
C6—C1—C2—C3	−1.2 (3)	C7—N1—C11—C10	−0.3 (3)
Sn1—C1—C2—C3	177.39 (15)	C9—C10—C11—N1	−0.1 (3)

Symmetry codes: (i) −*x*+1, −*y*+1, −*z*+1.

### Hydrogen-bond geometry (Å, °)

**Table:**

*D*—H···*A*	*D*—H	H···*A*	*D*···*A*	*D*—H···*A*
N1—H1···Br1	0.88	2.55	3.317 (2)	146
